# Interactive Effects of Dorsomedial Hypothalamic Nucleus and Time-Restricted Feeding on Fractal Motor Activity Regulation

**DOI:** 10.3389/fphys.2016.00174

**Published:** 2016-05-18

**Authors:** Men-Tzung Lo, Wei-Yin Chiang, Wan-Hsin Hsieh, Carolina Escobar, Ruud M. Buijs, Kun Hu

**Affiliations:** ^1^Medical Biodynamics Program, Division of Sleep and Circadian Disorders, Brigham and Women's Hospital, Harvard Medical SchoolBoston, MA, USA; ^2^Institute of Translational and Interdisciplinary Medicine and Department of Biomedical Sciences and Engineering, National Central UniversityTaoyuan, Taiwan; ^3^Departamento de Anatomía, Facultad de Medicina, Edificio “B” 4° Piso, Universidad Nacional Autónoma de MéxicoMéxico, Mexico; ^4^Departamento de Biología Celular y Fisiología, Instituto de Investigaciones Biomédicas, Universidad Nacional Autónoma de MéxicoMéxico, Mexico

**Keywords:** motor activity, food anticipation, fractal regulation, circadian rhythm, suprachiasmatic nucleus, dorsomedial hypothalamic nucleus

## Abstract

One evolutionary adaptation in motor activity control of animals is the anticipation of food that drives foraging under natural conditions and is mimicked in laboratory with daily scheduled food availability. Food anticipation is characterized by increased activity a few hours before the feeding period. Here we report that 2-h food availability during the normal inactive phase of rats not only increases activity levels before the feeding period but also alters the temporal organization of motor activity fluctuations over a wide range of time scales from minutes up to 24 h. We demonstrate this multiscale alteration by assessing fractal patterns in motor activity fluctuations—similar fluctuation structure at different time scales—that are robust in intact animals with *ad libitum* food access but are disrupted under food restriction. In addition, we show that fractal activity patterns in rats with *ad libitum* food access are also perturbed by lesion of the dorsomedial hypothalamic (DMH)—a neural node that is involved in food anticipatory behavior. Instead of further disrupting fractal regulation, food restriction restores the disrupted fractal patterns in these animals after the DMH lesion despite the persistence of the 24-h rhythms. This compensatory effect of food restriction is more clearly pronounced in the same animals after the additional lesion of the suprachiasmatic nucleus (SCN)—the central master clock in the circadian system that generates and orchestrates circadian rhythms in behavior and physiological functions in synchrony with day-night cycles. Moreover, all observed influences of food restriction persist even when data during the food anticipatory and feeding period are excluded. These results indicate that food restriction impacts dynamics of motor activity at different time scales across the entire circadian/daily cycle, which is likely caused by the competition between the food-induced time cue and the light-entrained circadian rhythm of the SCN. The differential impacts of food restriction on fractal activity control in intact and DMH-lesioned animals suggest that the DMH plays a crucial role in integrating these different time cues to the circadian network for multiscale regulation of motor activity.

## Introduction

One of the most important external stimuli that influence locomotor activity control is food availability. When food is provided to animals only during a fixed and limited time period each day (i.e., time-restricted feeding), animals will show food-anticipatory activity (FAA) after 5–7 days, which is characterized by increased activity levels (1–3 h) just before the feeding period (Mistlberger, 1994; Stephan, 2002; Silver et al., 2011). Once FAA is established, it will take several days before FAA completely disappears when food restriction is omitted, and it will reappear at the same time of the day when animals are food deprived again. FAA is believed to be controlled by an intrinsic food entrained oscillator or oscillatory network with a circadian period close to 24 h (Escobar et al., 2009). This is evident from the fact that FAA occurs only when the cycle of restricted food availability is close to 24 h or harmonics of 24 h such as 12 h (Mistlberger et al., 2012; Patton et al., 2014), e.g., FAA is absent when the cycle period is < 20 h (Boulos et al., 1980; Mistlberger and Marchant, 1995; Takasu et al., 2012). However, the evaluation of FAA has been exclusively focused on mean activity levels a few hours before and/or throughout the feeding period and on the circadian/daily rhythmicity of FAA. How time-restricted feeding affects dynamics of motor activity at different times of the daily cycle and at different time scales is largely unknown.

A wide range of biological functions from cellular processes to overt system behavior display endogenous circadian rhythms of ~24 h that are regulated by the circadian timing system, a network consisting of a huge number of cellular autonomous circadian clocks in the brain and peripheral organs (Weaver, 1998; Reppert and Weaver, 2002). In mammals, the master clock of the circadian system is located in the hypothalamic suprachiasmatic nucleus (SCN), which orchestrates circadian rhythms in behavior and physiology through numerous neural and humoral feedback loops (Sakamoto et al., 1998; Weaver, 1998; Yamazaki et al., 2000). In addition to circadian/daily rhythms in physiological functions, the SCN also impacts motor activity at different time scales from minutes to hours (Pittman-Polletta et al., 2013). Lesioning the SCN in animals not only abolishes circadian rhythm of motor activity but also disrupts the temporal organization of motor activity fluctuations that normally display fractal patterns—similar temporal structure and statistical properties at different time scales (Hu et al., 2007). Moreover, we found recently that interrupting the normal synchronization between endogenous circadian rhythms and behavioral cycles can also disrupt fractal activity patterns in animals with intact SCN, resembling the effect of the SCN lesion (Hsieh et al., 2014). Together these results provide strong evidence that the circadian control network plays an essential role in activity regulation at multiple time scales rather than only at a single time scale of ~24 h.

There is evidence for the mechanistic interactions between FAA and SCN-enforced circadian oscillations. For instance, circadian clock genes, such as Cry1, Cry2, and Bmal1, that are critical for the SCN function at neural level also play essential roles in regulating FAA rhythms (Takasu et al., 2012); and the SCN and its interaction with the dorsomedial hypothalamic (DMH) nucleus, another important neural node that influences motor activity though not generating circadian rhythms, contribute to the intensity of FAA (Gooley et al., 2006; Mieda et al., 2006; Angeles-Castellanos et al., 2010; Acosta-Galvan et al., 2011; Merkestein et al., 2014) though neither the SCN nor the DMH is required for the existence of FAA (Landry et al., 2006; Mistlberger et al., 2009; Moriya et al., 2009). In addition, we have shown that the SCN activity, as measured by vasopressin release or with c-Fos, is inhibited when animals exhibit FAA (Kalsbeek et al., 1998; Acosta-Galvan et al., 2011). Thus, it is possible that time-restricted feeding can affect the temporal structure of motor activity via its influences on SCN activity and the circadian system. Here we hypothesize that time-restricted feeding not only induces FAA but also affects motor activity control at multiple time scales, altering fractal activity patterns. We further hypothesize that the multiscale effect of time-restricted feeding on motor activity control is via its influence on the component in the circadian network underlying food entrainment. Thus, we test whether the effect of time-restricted feeding on fractal activity patterns is different in animals with the sequential lesions of the DMH and the SCN. Note that time-restricted feeding during the normal inactive phase induces a time cue “conflicting” to the endogenous circadian time of the SCN. For animals without the SCN, it is possible that time-restricted feeding can have different influences on multiscale activity regulation as compared to those in animals with the intact SCN. Specifically, we expect that time-restricted feeding in animals after the SCN lesion enhances daily activity rhythm and, thus, helps to restore disrupted fractal activity patterns in these animals.

## Materials and methods

### Ethics statement

All animal experiments were approved by the ethical committee at the Instituto de Investigaciones Biomédicas performed according to the guide for care and use of animal experimentation in Universidad Nacional Autónoma de México, which conforms to international guidelines for animal handling.

### Animals and protocols

To test our hypothesis, we studied locomotor activity of 18 Wistar rats that were placed in individual cages under 12:12 LD cycles. Animals were divided into three groups. (Group 1) Six rats (controls) were not disturbed with *ad libitum* food access for 2–4 weeks. (Group 2) Six rats underwent a 16-day scheduled food restriction protocol in which food was only available at Zeitgeber time (ZT) 6–8 h during the light phase. The experimental procedures were highly controlled to ensure that only food availability was the only different factor between the two experimental protocols for Group 1 (i.e., with *ad libitum* food access) and Group 2 (i.e., with food only available in a restricted period every day), respectively. (Group 3) The other six animals underwent two sequential surgeries for the lesions of DMH and SCN, respectively (see details below). After each surgery, animals went through the same protocol with *ad libitum* food access as the animals in Group 1 for at least 12 days and then the same 16-day food restriction protocol as the animals in Group 2.

### Lesions

For the DMH or SCN lesion, animals were anesthetized with 200 μL Ketamine (10 mg/100 g) and 165 μL Xilazine (1 mg/100 g) and mounted in a stereotaxic frame. Lesions were aimed to the DMH or the SCN with either kainic acid (a bilateral injection of 1% kainic acid, 100 nL Sigma) or with electrolytic current (a constant current of 0.2 μmAmp of 6V was used for 1 min bilaterally; Acosta-Galvan et al., 2011).

### Data acquisition

All data were previously collected and published (Angeles-Castellanos et al., 2010; Acosta-Galvan et al., 2011). Locomotion activity data were collected using motion sensors located at the bottom of cages that were designed to continuously monitor the animal motion via the vibration of the cages. Data were collected at 1-min intervals and were re-sampled with the epoch length of 4 min. To minimize transitional artifacts and training effects, data during the first 4 days after the lesion surgery and data during the first 7 days of each food restriction period were excluded from data analysis.

### Fractal analysis

To assess fractal activity regulation, we examined temporal correlations in activity fluctuations at an array of time scales n (from 0.1 to 20 h). We used the detrended fluctuation analysis (DFA) to quantify the fluctuation amplitude, F (n) at different time scale n (Peng et al., 1995). A power-law form of F (n) indicates fractal fluctuations, yielding F (n)~n^α^ (a straight line in a log–log plot). The parameter α (the scaling exponent) quantifies the correlation properties in fluctuations: α = 0.5 indicates no correlation (“white noise”); α > 0.5 indicates positive correlations. The most interesting, complex behavior is associated with α ~1.0 which indicates a fine balance between uncorrelated randomness and excessive regularity as observed in healthy physiological outputs (Stanley et al., 1992; Goldberger et al., 2002).

### Statistical analysis

Results are represented as mean ± standard error (SE). To assess the effect of time-restricted feeding on fractal activity patterns, ANOVA was performed under each condition (intact, the DHM lesion, and the lesions of both the DMH and the SCN). We compared the results of Group 1 and Group 2 (i.e., between-subjects comparison) to determine the effect of time-restricted feeding in animals with intact DMH and SCN. We compared the results of the same animals in Group 3 under two experimental protocols (i.e., within-subjects comparison) to determine the effect of food restriction in animals after lesioning the DMH or after lesioning the SCN. To study the effect of the lesions, ANOVAs were used to determine the difference among the three conditions for *ad libitum* and time-restricted feeding, separately. Similar ANOVAs were also performed to assess the effect of food restriction and the lesions on the amplitude of daily activity rhythm.

## Results

### Time-restricted feeding induced FAA and disrupted fractal regulation in intact animals

**Figure 1:**
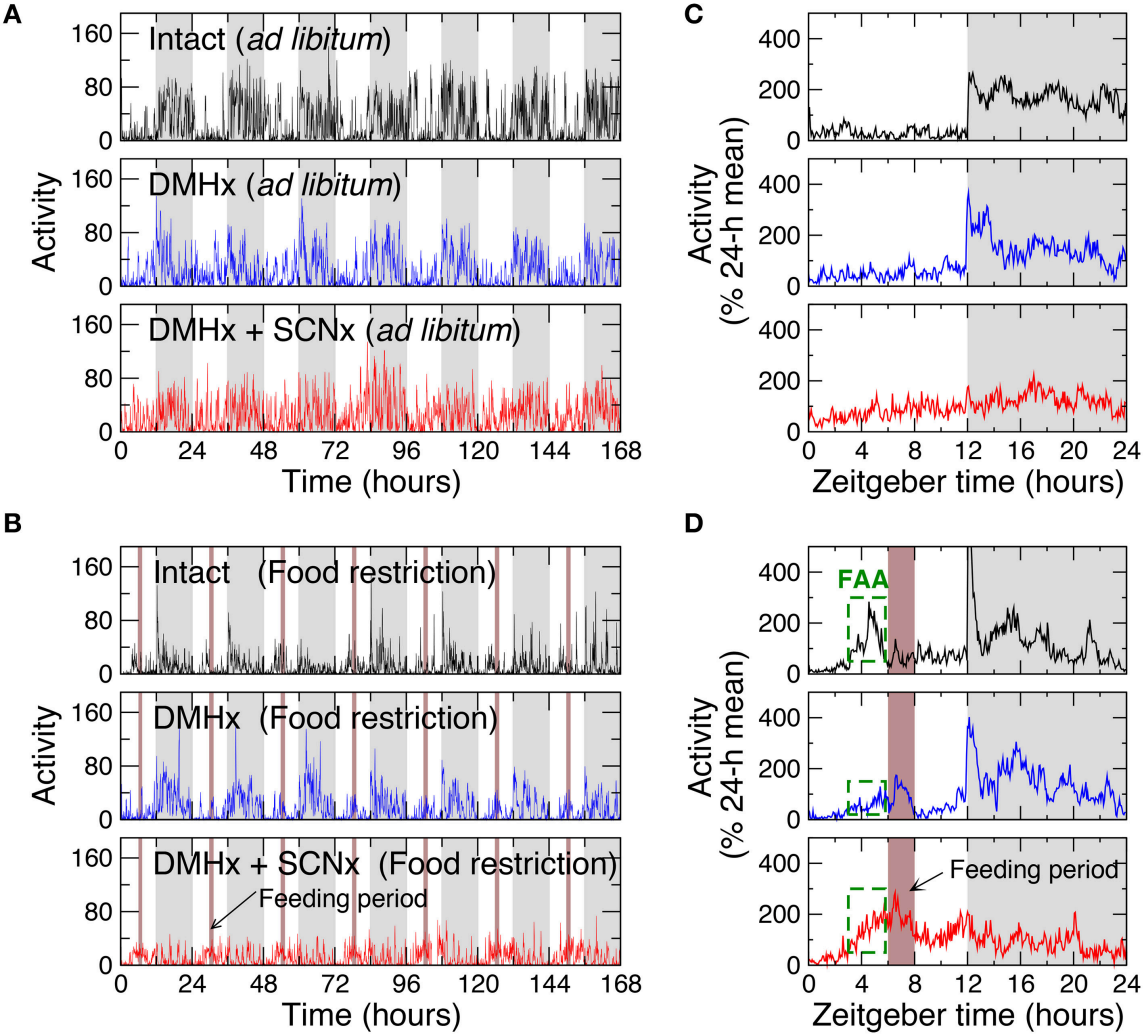
**Representative locomotor activity recordings and their averaged daily profiles of rats under 12 h:12 h light-dark cycles. (A)** Recordings of an intact rat, a rat with the lesion of DMH (DMHx rat), and the same DMH-lesion rat after an additional lesion of the SCN with *ad libitum* food access. Gray (white) bars indicate the dark (light) periods of the light-dark cycle. **(B)** Recordings of an intact rat and a DMHx rat (the same as in **A**) before and after the SCN lesion with restricted food availability. Food was available daily for 2 h at 6–7 h after light on in each cycle (brown bars). **(C)** The averaged 24-h activity waveforms of the recordings in (**A)**. **(D)** The averaged 24-h waveforms of the recordings in (**B)**. To obtain the 24-h activity patterns, data were first normalized by daily means and then averaged across different days. The increased activity levels 3 h before the feeding period (green dashed boxes) indicate food-anticipatory activity (FAA).

We first examined the influences of time-restricted feeding on intact animals by studying 6 rats with *ad libitum* food access and 6 rats undergoing a food restriction protocol with a 2-h feeding period at 6–8 h after lights on (Zeitgeber time: ZT 6–8; Figures 1A,B). With *ad libitum* food access, animals showed a 24-h rhythm in locomotor activity with low levels during the 12-h light phase (44 ± 4% of daily mean) and high levels during the 12-h dark phase (156 ± 4% of daily mean; *p* < 0.0001; Figure 1C). Food restriction induced FAA, as characterized by significantly increased activity levels starting 3 h before the feeding period (at ZT 3–6, food restriction: 141 ± 4% of daily mean; *ad libitum*: 45± 6% of daily mean; *p* < 0.0001) as well as the mean activity level during the entire light phase (80 ± 3% of daily mean; *p* < 0.0001; Figure 1D). Such increased activity during the light phase caused an overall reduced 24-h rhythm.

**Figure 2:**
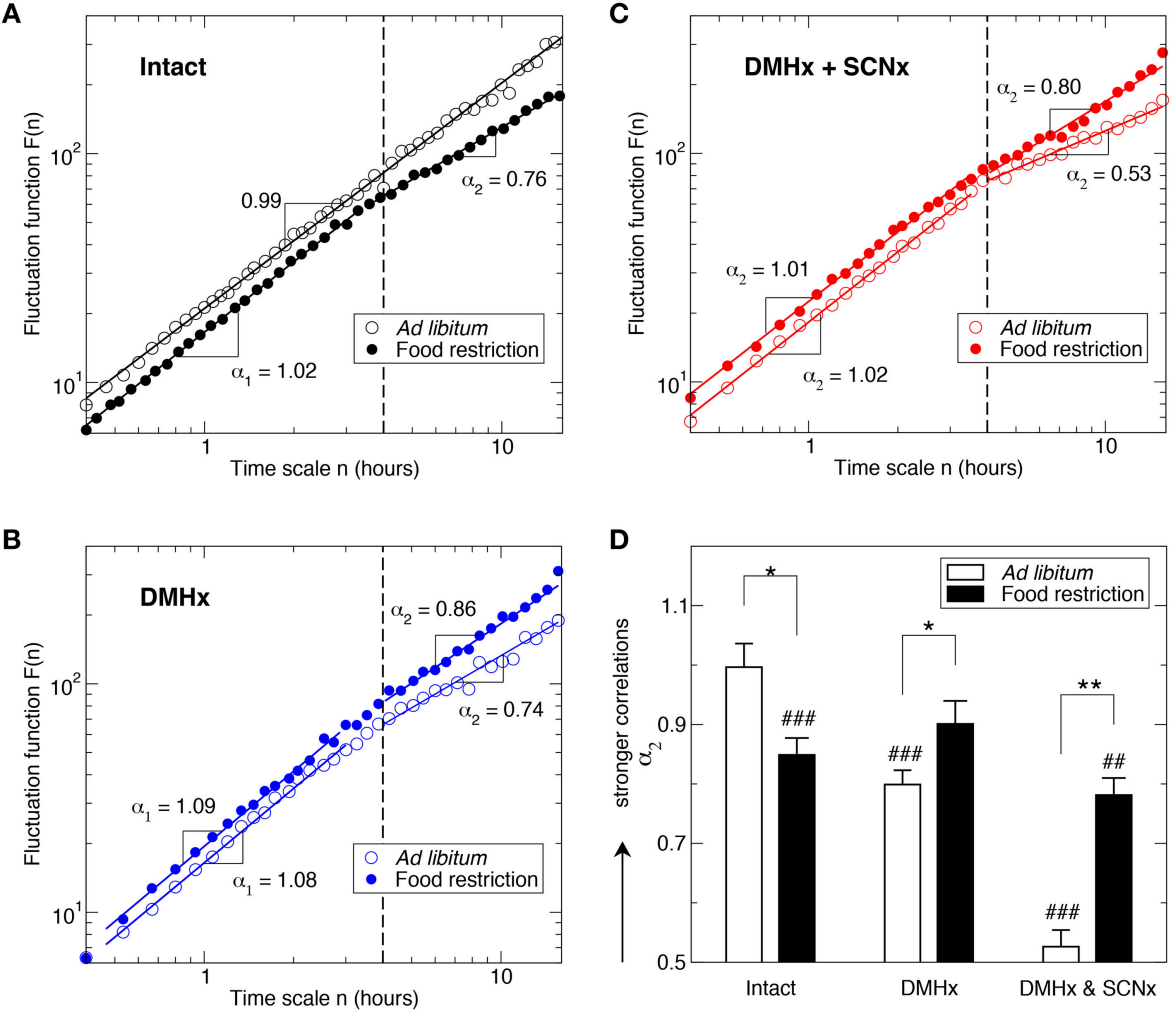
**Influence of food restriction on fractal activity patterns in intact animals and in animals after the lesions of the SCN and the DMH**. Fluctuation functions of intact animals **(A)**, a rat after the DMH lesion **(B)**, and the same rat (as in **B**) after the lesions of the DMH and the SCN **(C)**. Data are shown on log-log plots. Fluctuation functions are vertically shifted for a better visualization of the influence of food restriction. Results were obtained from the signals shown in Figure 1 using the detrended fluctuation analysis. **(D)** Scaling exponents at time scales > 4 h. ^*^ indicates the effect of food restriction: ^*^p ≤ 0.02 and ^**^p ≤ 0.002. ^#^indicates the difference between large (>4 h) and small time scales (<4 h): ^#^*p* ≤ 0.02, ^*##*^*p* ≤ 0.002 and ^*###*^*p* ≤ 0.0002.

In addition to the 24-h rhythm, activity in the intact animals with *ad libitum* food access exhibited robust fractal fluctuations as characterized by a power-law form of F(n) ~ n^α^ that persisted across a broad range of time scales from minutes up to 12 h (Figure 2A). Consistent with the previously reported (Hu et al., 2007; Hsieh et al., 2014), the scaling exponent (α = 0.99 ± 0.02) was close to 1, indicating a complex structure with strong temporal correlations in activity fluctuations. Markedly, time-restricted feeding perturbed fractal activity patterns, leading to different correlations in activity fluctuations over two time-scale regions as indicated by the different scaling exponent (*p* = 0.0002): α_1_ = 1.10 ± 0.02 at time scales < ~4 h (Region I) and α_2_ = 0.85 ± 0.03 at time scales > ~4 h (Region II; Figure 2A). As compared to the results without food restriction, correlations were stronger in Region I (i.e., larger α_1_; *p* = 0.0008) but were much weaker in Region II (i.e., smaller α_2_, *p* = 0.013; Figure 2D). The difference in correlations between two regions remained when excluding the data before and throughout the feeding period (ZT 3–8 h; *p* = 0.0085).

### DMH lesion reduced FAA, disrupted fractal regulation, and reversed the effect of food restriction on fractal regulation

To test whether the DMH is involved in fractal activity regulation and plays any role in the influence of time-restricted feeding on multiscale activity patterns, we then studied locomotor activity recordings of 6 rats after the lesion of the DMH with *ad libitum* food (Figure 1A). As compared with intact rats, the DMH-lesioned (DMHx) animals maintained the 24-h activity rhythm, showing no significant differences in activity level during the light phase (ZT0–12 h: DMHx: 44 ± 4% of daily mean; intact: 44 ± 4% of daily mean; *p* > 0.9; Figure 1C). As occurred in the intact animals, food restriction also increased activity levels during the light phase (56 ± 4% of daily mean; *p* = 0.0068) and led to an overall reduced circadian rhythmicity in DMHx animals (Figure 2D). However, the responses to food restriction in these DMHx animals were much weaker than those in the intact animals, as indicated by the lower activity level during the light phase (*p* = 0.0009). In addition, the increase in the mean activity level at ZT 3–6 (3 h before the feeding time) did not reach a significant level in these DMHx animals (*ad libitum*: 35 ± 2% of daily mean; food restriction: 45 ± 4% of daily mean; *p* = 0.11). Together these results confirmed a reduced FAA in these DMHx animals as previously reported (Gooley et al., 2006; Acosta-Galvan et al., 2011).

Despite the persistence of 24-h activity rhythmicity, lesioning the DMH significantly disrupted fractal activity patterns in a similar manner as the food restriction did to intact animals (Figures 2A,B), i.e., increasing the correlations at time scales < ~4 h (Region I: α_1_ = 1.06 ± 0.02; *p* = 0.0093) while reducing the correlations at time scales > ~4 h (Region II >: α_2_ = 0.79 ± 0.04; *p* = 0.0041). Surprisingly, food restriction had different effects on activity correlations in these DMHx animals as compared to intact animals, i.e., instead of disrupting fractal patterns, food restriction reduced the difference in the correlations between Region I and Region II such that the scaling exponents in the two regions became not significantly different in the DMHx animals (Region I: α_1_ = 1.04±0.03; Region II; α_2_ = 0.90 ± 0.04; *p* = 0.08; Figure 2D). Such “rescuing” effect of food restriction was more pronounced after excluding the data before and throughout the feeding period (ZT 3–8), i.e., α_1_ and α_2_ became virtually the same (α_1_ = 1.05 ± 0.03; α_2_ = 1.05 ± 0.03; *p* > 0.9), suggesting fully restored fractal activity patterns.

### Additional lesion of the SCN improved FAA and enhanced the effect of time-restricted feeding on fractal regulation

We further explored the role of the SCN in the influence of time-restricted feeding on motor activity regulation by studying the same 6 DMH-lesioned animals after the additional lesion of the SCN (Figures 1A,B). As expected, lesioning the SCN significantly diminished the 24-h activity rhythm as characterized by a lower normalized power density at 24 h (0.07 ± 0.02; *p* = 0.0004; Figure 1C). Note that the remained 24-h activity rhythm was likely due to the masking effect of the LD cycles. With the additional lesion of the SCN, time-restricted feeding induced pronounced FAA as indicated by the increased activity level at ZT 3–6 (food restriction: 158 ± 10% of daily mean; *ad libitum*: 85 ± 7% of daily mean; *p* = 0.0012). FAA reversed the 24-h active-inactive rhythm, leading to higher activity level during the light phase (ZT 0–12: 106 ± 5% of daily mean, *p* = 0.0024) than the level during dark phase (Figure 1D).

Lesioning the SCN further disrupted the fractal activity patterns in these animals (Figure 2C). Specifically, activity fluctuations at time scales > ~4 h became more random as indicated by a much smaller α_2_ (0.53 ± 0.03; *p* < 0.0001; Figure 2D). Consistent with our previous observation in rats with only the lesion of the SCN (Hu et al., 2007), the mean value of α_2_ was not significantly different from 0.5 (*p* > 0.3), suggesting the white-noise type of fluctuations without any correlations at >~4 h. The correlations in Region I (α_1_ = 1.06 ± 0.02) remained the same as those before the SCN lesion (*p* > 0.4).

Similar to that observed in these animals before the SCN lesion, time-restricted feeding reduced the difference in correlations between Region I and Region II in the same animals after the SCN lesion (Figures 2C). The main effect was on the correlations in Region II (α_2_ = 0.78 ± 0.04; *p* = 0.0015 for the comparison with *ad libitum* food). The food-induced increase in α_2_ was greater than the change for these animals with only the lesion of DMH (after the SCNx: Δα_2_ = 0.25; before the SCNx: Δα_2_ = 0.11). Despite the “rescuing” effect of time-restricted feeding, α_2_ was still significantly smaller than α_1_ (1.03 ± 0.01; *p* = 0.0006; Figure 2). The correlations in Region I were not significantly affected by food restriction (*p* > 0.3).

## Discussion

### Food restriction impacts multiscale activity control

Recurring feeding in a fixed, short period during the light phase is widely used to study the anticipatory behavior in animals. However, previous studies have exclusively focused on the change of mean activity levels 1–3 h before the feeding period in each 24-h activity cycle. By examining the temporal structure in activity fluctuations, we showed in this study that food restriction with 2 h of food feeding each day not only induced food anticipatory activity but also disrupted dynamics of motor activity across the entire 24-h cycle and over a wide range of time scales from minutes up to 12 h. The disruption is characterized by the breakdown of fractal patterns in activity fluctuations that were robust in intact animals with *ad libitum* food access. Importantly, the disruption resembles those observed in animals after the lesion of the SCN (Hu et al., 2007) and in humans with dementia and/or Alzheimer's disease (Hu et al., 2009, 2013), providing strong evidence that time-restricted feeding during the inactive phase has an adverse impact on motor activity control in intact animals at multiple time scales.

**Figure 3:**
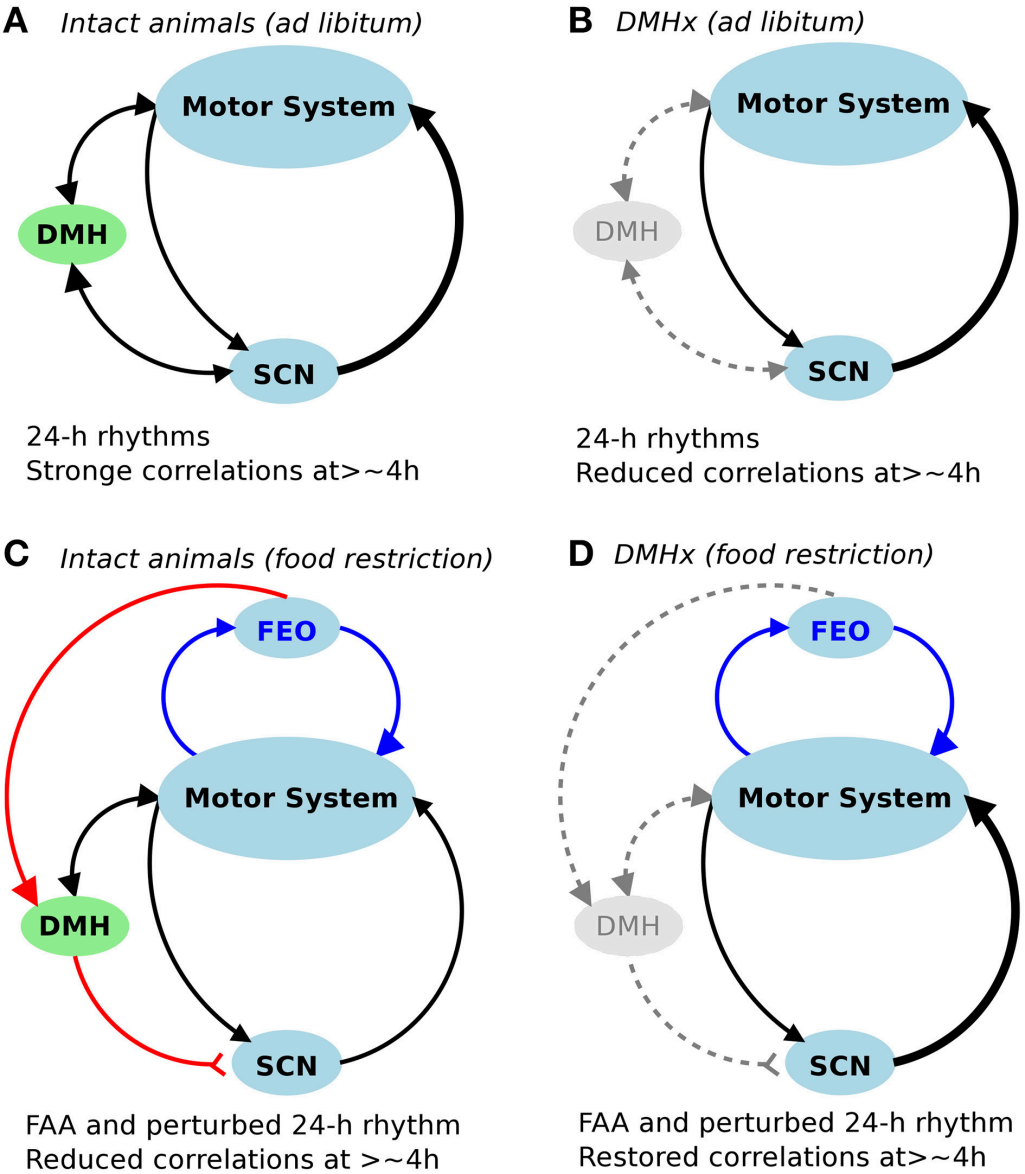
**Schematic diagram of the control network for fractal activity regulation**. **(A,B)** In addition to the SCN, the DMH is required for fractal activity regulation in intact rats with free access to food. Lesioninng the DMH led to disrupted fractal regulation as characterized by significantly reduced correlations at >4 h **(B)**. **(C,D)** Restricted food access during the normal inactive periods (light phase of the light-dark cycles) had opposite impacts on fractal activity regulation in intact animals (i.e., causing a reduction in correlations at >4 h) and in DMH-lesioned animals (i.e., causing an increase in correlations at >4 h), despite the similar influences on 24-h activity rhythms (i.e., food-anticipatory activity and reduced amplitude of 24-h rhythm).

Fractal patterns have been demonstrated in many neurophysiological signals such as heartbeat, gait, respiration, motor activity, and brain activity (Peng et al., 1995, 2002; Hausdorff et al., 1996; Ivanov et al., 2007; Hu et al., 2008a,b; Anteneodo and Chialvo, 2009; He et al., 2010; Fraiman and Chialvo, 2012). One influential interpretation of these fractal physiological fluctuations is the existence of a “long-term memory” in the control system (Stanley et al., 1992; Goldberger et al., 2002; West, 2010a,b). Intuitively, the circadian timing system should participate in memory-keeping because it is responsible for coordinating circadian rhythms in many physiological functions and processes in synchrony with the light-dark cycles (Sakamoto et al., 1998; Weaver, 1998; Yamazaki et al., 2000). Disrupting these circadian oscillations/rhythms and their synchronization, as occurs with aging and in shift work, can lead to deleterious physiological consequences, such as sleep disorders and increased risk for diabetes and cardiovascular diseases (Knutsson et al., 1986; Kawachi et al., 1995; Penev et al., 1998; Schwartz and Roth, 2006; Kroenke et al., 2007; Martino et al., 2007). Thus, the observed disruption in fractal activity patterns under time-restricted feeding may reflect disturbed circadian timing regulation due to two conflicting inputs for circadian timing: one from the light-dark cycle and the other one from the cycle of food-related stimulus (Figure 3). This hypothesis is also consistent with our recent finding that forced activity during the habitual resting phase (light phase: ZT 2–10) could also disrupt fractal activity regulation (Hsieh et al., 2014). Further studies are warranted to test/clarify this theory of memory keeping and circadian timing.

### Fractal regulation involves both the SCN and the DMH

It is worth noting that fractal patterns and circadian rhythms represent different properties of the motor control system. Fractal regulation requires an integrated network of multiple control nodes with feedback interactions (Chialvo, 2010). Though the SCN is a major node in the network for fractal activity control, our previous study showed that the neural network within the SCN cannot generate fractal patterns since the neural activity fluctuations of the SCN *in vitro* display no fractal patterns (Hu et al., 2012). In this study we confirm the role of the SCN in fractal activity regulation. We also show that fractal activity patterns can be significantly disrupted despite the persistence of the 24-h/circadian rhythms as occurred in the animals after the DMH lesion. These results showed that lesions of the DMH had similar disruptive effects on fractal activity patterns as compared to lesioning only the SCN, leading to more random fluctuations at time scales > ~4 h and more regular fluctuations at smaller time scales. These results provide direct evidence that the DMH is also important for the maintenance of fractal activity control (Figure 3A). We note that activity fluctuations in the DMH-lesioned animals still possess certain correlations at time scales >~4 h (indicated by α_2_ > 0.5), which were completely abolished after the additional lesion of the SCN. These observations suggest that the SCN imparts fractal activity control also through neural nodes other than the DMH (Figure 3B). This hypothesis is supported by the fact that circadian rhythms in physiological variables, including motor activity and temperature, persist after the DMH lesion.

### Effect of food restriction on fractal regulation

The most intriguing observations in this study are that food restriction partially rescued the disrupted fractal activity patterns in animals after the lesions of DMH and SCN, and almost fully restored fractal activity in the DMH-lesioned animals (Figures 2A,B). These results have two implications with regard to FAA and fractal activity control.

First, these results suggest that, in addition to SCN-related interactions, there may be food-triggered redundant feedback interactions for the maintenance of fractal activity regulation. Hypothetically these interactions are relevant to the neural circuitry of FAA. It is established that FAA rhythms are only present in feeding schedules with periodicity in the circadian range (Stephan, 1981). Since the SCN is not required for FAA, a separate food-entrained oscillator (FEO) is believed to exist within the circadian system. One previous hypothesis was that the FEO is located within the DMH (Chou et al., 2003; Gooley et al., 2006; Mieda et al., 2006) but this hypothesis has been refuted by many other studies, showing persisting FAA after lesioning the DMH (Landry et al., 2006; Mistlberger et al., 2009; Moriya et al., 2009). Despite the current limited understanding of FAA, our results provide evidence that the neural circuitry of FAA may be a key component in the control network for fractal activity regulation at multiple time scales (Figures 3C,D).

Moreover, these results seem to contradict the results from the intact animals (i.e., food restriction disrupted fractal patterns). One possible explanation for this seemingly paradox is that the DMH serves as a “switch,” controlling how the information inputs from food restriction and from the light-dark cycles are integrated for fractal activity control. This reminds the role of the DMH in FAA. As we reported in a previous study (Acosta-Galvan et al., 2011) and also showed in this study, lesioning the DMH led to a suppression of FAA while subsequent lesioning the SCN diminished or abolished the suppression. Taken together, the DMH may serve as a double switch for the effects of food restriction on FAA and on fractal activity control: (1) the switch was normally on in intact animals to allow better food anticipation (when food is only available during the inactive phase) while sacrificing other circadian-related processes that are related to fractal activity control (Figure 3C); (2) lesioning the DMH turned off the switch, reducing FAA but allowing the circadian network to integrate the information inputs of food stimulus and of light-dark cycles for better fractal activity control (Figure 3D); and (3) when the SCN was also ablated, the timing of the circadian oscillation was lost such that time-restricted feeding induced more pronounced FAA and improved fractal activity control due to the lack of conflicting or competing time inputs from the SCN. Note that the “conflicting time inputs” could not explain the rescuing effect of time-restricted feeding in the animals after the DMH lesion (but with intact SCN) because daily/circadian rhythms persisted after the DMH lesion. Clearly future studies are required to determine the pathway through which the DMH controls the effect of time-restricted feeding on fractal activity regulation.

## Limitations

This is the first study demonstrating how time-restricted feeding affects fractal activity regulation. There are a number of notable limitations in this pilot study. First, during the protocol with time-restricted feeding, food was provided at 6–8 h after lights on during the light-dark cycles (i.e., ZT 6–8 h) when animals were normally inactive. Whether restricting feeding at different times can have different impacts on temporal activity correlations is yet to be determined. Specifically, if restricted food is available during the active period or dark phase, there should be no conflicting time cues for activity such that time-restricted feeding may enhance the endogenous circadian activity rhythm, thus not disrupting fractal activity control in control animals while better improving the multiscale control in DMH or SCN-lesioned animals. Second, we focused on fractal activity patterns and did not examine the rhythms at specific time scales except for 24-h rhythm. It is established that rodents exhibit ultradian feeding rhythms of 2–3 h (Gerkema et al., 1993; Bloch et al., 2013). Since the effect of the time-restricted feeding on temporal activity correlations was only pronounced at time scales >~4 h, the altered fractal patterns should be not related to potential changes in ultradian feeding rhythms. Third, to examine fractal regulation, we examined only temporal correlations. There are many different statistical properties such as nonlinearity and multifractality that may reveal different aspects of multiscale activity control (Ivanov et al., 1999; Ashkenazy et al., 2001). Finally, many different methods can assess temporal correlations and their performances may be comparable or even better than that of the detrended fluctuation analysis (Xu et al., 2005; Makarava et al., 2011). Thus, follow-up studies using different analytical tools are necessary to confirm and explain our observations.

Despite the limitations, this study revealed a previously un-recognized influence of time-restricted food availability on multiscale activity control. Our results provide solid evidence that inducing FAA does not simply impose an event (i.e., feeding period) at a specific circadian time but requires an adjustment in dynamic control of motor activity across the whole circadian cycle and at different time scales. The influence is likely via the impact of food stimulus as an additional time cue (in addition to light inputs) on the circadian network. The different effect of food restriction in the intact animals and in the DHM-lesioned animals indicates that the DMH plays a crucial role in integrating the timing information from both the SCN and food stimulus. Further studies are warranted to elucidate the interactions among the food-entrained oscillator, the DMH, and the SCN, as well as their mechanistic links to fractal activity regulation.

## Author contributions

ML, CE, RB, and KH conceived and designed the experiments. RB and CE performed the experiments and collected data. ML, WC, WH, and KH performed the data analyses. ML and KH provided analysis tools. ML, CE, RB, and KH wrote the manuscript.

### Conflict of Interest Statement

The authors declare that the research was conducted in the absence of any commercial or financial relationships that could be construed as a potential conflict of interest.
